# Efficient Cargo Delivery into Adult Brain Tissue Using Short Cell-Penetrating Peptides

**DOI:** 10.1371/journal.pone.0124073

**Published:** 2015-04-20

**Authors:** Caghan Kizil, Anne Iltzsche, Alvin Kuriakose Thomas, Prabesh Bhattarai, Yixin Zhang, Michael Brand

**Affiliations:** 1 German Centre for Neurodegenerative Diseases (DZNE) Dresden within the Helmholtz Association, Arnoldstr. 18, 01307, Dresden, Germany; 2 DFG-Center for Regenerative Therapies Dresden (CRTD) – Cluster of Excellence, Technische Universität Dresden, Fetscherstr. 105, 01307, Dresden, Germany; 3 B-CUBE, Center for Molecular Bioengineering, Technische Universität Dresden, Arnoldstr. 18, 01307, Dresden, Germany; 4 Biotechnology Center of the TU Dresden, Technische Universität Dresden, Tatzberg 47, 01307, Dresden, Germany; Hungarian Academy of Sciences, HUNGARY

## Abstract

Zebrafish brains can regenerate lost neurons upon neurogenic activity of the radial glial progenitor cells (RGCs) that reside at the ventricular region. Understanding the molecular events underlying this ability is of great interest for translational studies of regenerative medicine. Therefore, functional analyses of gene function in RGCs and neurons are essential. Using cerebroventricular microinjection (CVMI), RGCs can be targeted efficiently but the penetration capacity of the injected molecules reduces dramatically in deeper parts of the brain tissue, such as the parenchymal regions that contain the neurons. In this report, we tested the penetration efficiency of five known cell-penetrating peptides (CPPs) and identified two– polyR and Trans – that efficiently penetrate the brain tissue without overt toxicity in a dose-dependent manner as determined by TUNEL staining and L-Plastin immunohistochemistry. We also found that polyR peptide can help carry plasmid DNA several cell diameters into the brain tissue after a series of coupling reactions using DBCO-PEG4-maleimide-based Michael’s addition and azide-mediated copper-free click reaction. Combined with the advantages of CVMI, such as rapidness, reproducibility, and ability to be used in adult animals, CPPs improve the applicability of the CVMI technique to deeper parts of the central nervous system tissues.

## Introduction

The adult zebrafish brain has extensive adult neurogenesis and regeneration capacity, which mostly relies on proliferating progenitor cells located in distinct stem cell niches throughout the brain [1–3]. Understanding the molecular mechanisms underlying neural regeneration bears great potential for therapeutic applications. This demands tools for efficient analysis of gene function. Methods that are available for such uses in zebrafish include transgenic misexpression methods using driver lines with direct promoter fusions, Cre/lox [4] or Tet-On/Off-based conditional expression strategies [5], manual delivery of plasmids after electroporation [6], and cerebroventricular microinjection (CVMI) of antisense oligonucleotides, ligands or viruses [7–9]. These approaches are very valuable, yet have limitations such as time-consuming generation of transgenics, mosaic targeting after electroporation, or low penetration depth of morpholinos into the brain tissue. Hence drugs and functional agents often do not reach target areas located deep in the tissue. This remains a major obstacle given that the efficient delivery and targeting range is crucial for the success of function-blocking reagents. Here, we describe a novel delivery method, given that the crossing of biological barriers is one of the main obstacles that remains to be overcome for cell targeted therapies and functional studies.

Recently, cell-penetrating peptides (CPPs) have been identified as potent in vitro and in vivo delivery tools [10]. These are short and highly cationic peptides capable of delivering biological cargos across the plasma membrane [11]. Upon initial discovery in 1988 of the translocation activity of the HIV-1 Tat transactivation protein, CPP-mediated delivery gained rapid interest of the research society [12,13]. Schwarze et al. (1999) showed that the translocation activity of Tat49-57 can be exploited by coupling it to ß-galactosidase [14]. Upon coupling, the 120 kDa hydrolase enzyme was able to translocate across the blood-brain barrier when injected intraperitoneally. Additionally, Tat and other CPPs have been used for example for the delivery of proteins [15], peptides [16,17], nanoparticles [18], oligonucleotides [19] and siRNA [20–22]. Cerebroventricular microinjection (CVMI) as described in [7,8] provides an efficient way of delivering the peptides by injecting them into the cerebroventricular space of the adult zebrafish brain. An incision is created above the optic tectum through which the desired molecules are injected. High pressure injection leads to an even dispersion throughout the brain targeting the ventricular area along the entire rostrocaudal axis. In this report, we identify that an arginine rich delivery peptide (PolyR) shows strong penetration into almost 70% of the deep parenchymal tissue of the adult zebrafish brain with no overt cytotoxic effect or change in homeostatic stem cell proliferation upon administration. In addition, we find that PolyR can carry along small molecules easily while larger cargo molecules such as plasmid DNA result in less efficient, yet still significantly improved delivery into the deeper brain tissues. Therefore, the combination of CVMI and CPPs in one delivery method potentially allows delivery to tissues for which manual targeting was previous not possible. The use of peptides as delivery tools allows a broad targeting of cells deep within the parenchyma in thick tissues such as the brain. In combination with CVMI, peptides and their cargo can be delivered into the adult brain in zebrafish, making the use of these peptides robust and efficient delivery tags, not only for the central nervous system, but potentially also for other tissues.

## Results and Discussion

### 5-Carboxyfluorescein-labeled peptides penetrate the brain tissue

**Fig 1:**
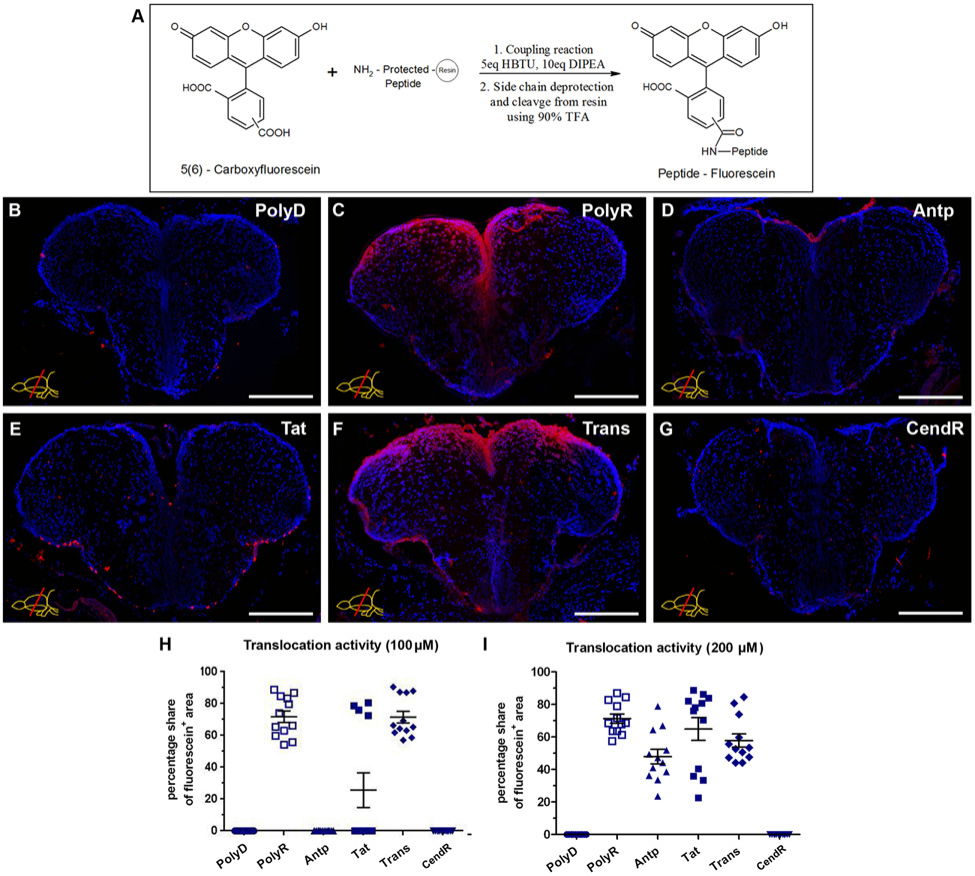
Overview of Peptide Translocation in the Telencephalon upon 100 μM Injection, and Dose-dependence. (A) Reaction scheme for the coupling of 5(6)-carboxyfluorescein to peptides. Fluorescein immunohistochemistry (IHC) on rostral telencephalon of peptide (B) control PolyD, (C) PolyR, (D) Antp, (E) Tat 49–57, (F) Trans and (G) CendRP injected brains. Graphs depict the average area of peptide translocation in one telencephalic hemisphere, upon 100 μM dose injection (H) and upon 200 μM dose injection of CPPs (I). Scale bars: 200 μm, n = 3, data are mean ± s.e.m.

In order to identify peptides that successfully enter deep into the brain tissue, we synthesized five cell penetrating peptides using standard Fmoc solid-phase peptide synthesis chemistry [23–25] (Table 1). Poly-Aspartic Acid (PolyD) was designed as an anionic control peptide that would not penetrate the tissue in contrast to the cationic amino acid residues, which are known to be crucial for the peptide trafficking [26]. Negatively charged compounds are often very difficult to penetrate cell membrane. For example, it is notoriously difficult to deliver phosphorylated peptide or oligonucleotides into cells. The poly-D possesses many negative charges, which prevents its interaction with phospholipid membrane. This concept has been used to prevent drug from entering into cells [27] or to carry out chemical reaction outside cells [28]. Therefore, we used polyD as a control peptide. Poly-Arginine (PolyR) [11], Antennapedia (Antp) [11,29], basic nuclear localization sequence containing region of the transcriptional activator region of the HIV Tat protein 49–57 (Tat_(49–57)_) [14,17,30,31], Transportan (Trans) [11,32] and C-end rule (CendR) peptide (CendRP) [33,34] were selected based on previous in vivo and in vitro translocation success. To allow the detection of peptides in the tissue, 5(6)-carboxyfluorescein was coupled to the N-terminus of these peptides. (Fig 1A).

**Table 1:** Synthesized Peptides used for CVMI-based Translocation Experiments.

Name	Sequence
PolyD	DDDDDDDDD
PolyR	RRRRRRRRR
Antp	RQIKIWFQNRRMKWKK
Tat_(49–57)_	RKKRRQRRR
Trans	GWTLNSAGYLLGKINLKALAALAKKIL
CendRP	RPARPAR

Shown are the name and sequence of the peptides. All peptides were synthesized using standard FMOC chemistry and coupled to 5-Carboxyfluorescein to allow detection in the tissue.

**Fig 2:**
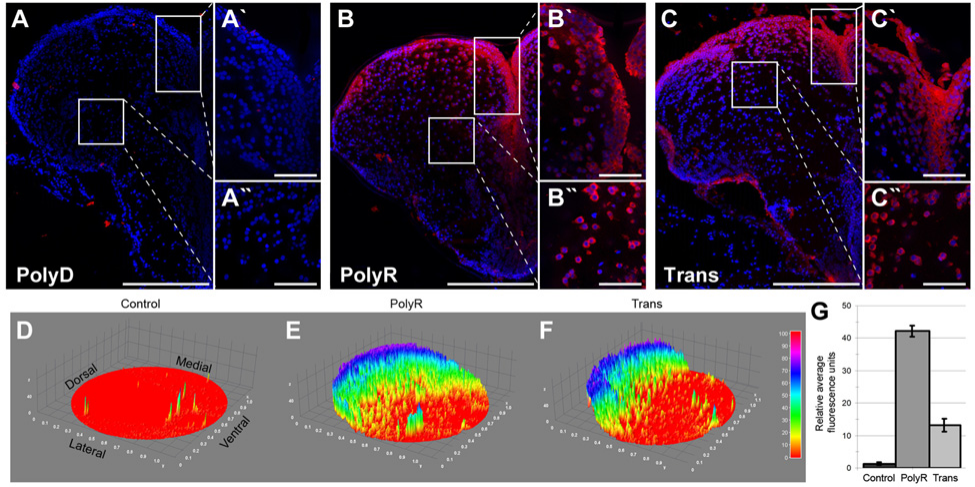
High Magnification Images of the Translocation of the Peptides PolyR and Trans with Surface Plots. Fast-Red staining of cryosections upon 100 μM dose injection of peptides. (A) Control peptide PolyD does not translocate; neither into the ventricular area (A') nor parenchyma (A''). (B) PolyR shows strong translocation in the ventricular area (B') yet also into the parenchyma (B''). (C) Brain section showing Trans localization in ventricular area (B') and parenchyma (C''). Scale bars: 200 μm in A, B, C overviews; 50 μm in A, B, C insets.

After CVMI of fluorescein-coupled peptides into the brain and immunohistochemical detection of fluorescein at 1 day post injection (dpi) (Fig 1B–1G) we observed that the peptides PolyR and Trans penetrated into an average area of 70 ± 4% of each telencephalic hemisphere upon an injection concentration of 100 μM (Fig 1C, 1F and 1H; Fig 2A–2C”), whereas the control peptide and the peptides PolyD, Antp and CendRP did not show any penetration (Fig 1B, 1E, 1G and 1H; Fig 2A–2A”). Tat spread into 27 ±13% of the telencephalon in only one of the three injected brains in an inconsistent manner (Fig 1D and 1H). To determine the level of peptides translocated to the deep tissues, we quantified the relative fluorescence levels in the telencephalic hemispheres, and observed that compared to no translocation of the control PolyD (Fig 2D and 2G), PolyR (Fig 2E) and Trans (Fig 2F) showed significantly elevated levels of penetration, with PolyR being 3-fold more effective than Trans (Fig 2G). These results indicate that PolyR and Trans peptides are readily and consistently penetrating into deep brain tissues in adult zebrafish upon injection at 100 μM concentration.

### Penetration capacities of peptides are dose-dependent

It has been shown that the dose of the administered peptides is crucial for translocation activity, yet higher doses increase the risk of cytotoxic side effects upon tissue entry [35]. To identify a potential dose-dependence on penetration depth, we injected a higher dose of 200 μM. At this concentration, PolyR translocation remains at an average 70 ± 5%, whereas Trans shows a modest decrease to 58 ± 6% (Fig 1I). The consistency of Tat translocation increased upon 200 μM dose injection, yet the variance remains high, showing an average translocation of 63 ± 11% (Fig 1I). These results indicate that PolyR and Trans peptides already reach their maximum penetration capacity at a concentration of 100 μM, and the low consistency and the high doses required for efficient penetration limits the applicability of Antp and Tat (Fig 1H). Additionally, the peptides PolyR and Trans proved to be promising candidates for tissue penetration and potentially concomitant cargo delivery into the deep brain tissue in adult zebrafish brain. Therefore, we selected the peptides PolyR and Trans, as well as PolyD as a negative control, for subsequent analyses.

### Trans but not the PolyR peptide induces cytotoxic side-effects upon translocation at high doses

**Fig 3:**
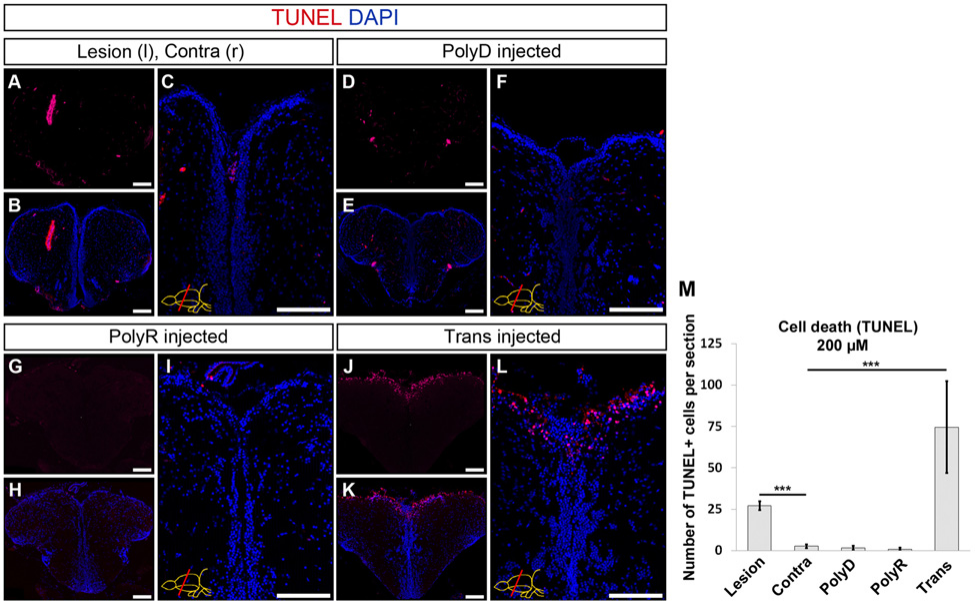
Quantification of Cell Death upon Peptide Injection. (A) TUNEL staining on rostral telencephalon of stab-lesioned brain, used as control. Lesioned hemisphere left and unstabbed contralateral hemisphere right. (B) DAPI counterstaining on A. (C) Magnified medial ventricular region of B. (D) TUNEL upon PolyD injection and DAPI counterstaining (E). (F) High magnification of medial ventricular region of E. (G) TUNEL staining on PolyR-injected brain and DAPI counterstaining (H). (J) TUNEL on Trans-injected brain. (K) DAPI counterstaining on J and high magnification of medial ventricular region (L). (M) Quantification of TUNEL-positive cells per telencephalic hemisphere. Scale bars = 100 μm, n = 3, data are mean + s.e.m.

Since the peptides perturb the cell plasma membrane are foreign material that is taken up by the cells, they might cause cytotoxic effects, which would strongly limit their use as delivery tools. Therefore, we analyzed whether PolyR and Trans cause cell death upon injection to the zebrafish brain at the maximum tested concentration of 200 μM. We quantified the number of apoptotic cells at 1 dpi using TUNEL assay (Fig 3). To allow comparison, a stab-lesioned brain (24 h post lesion) was used as a positive control, which gives an average of 27 ± 4 TUNEL-positive cells in the stabbed hemisphere as opposed to 2 ± 1 cells in the unstabbed contralateral hemisphere per section (Fig 3A–3C and 3M). Injection of control PolyD peptide at 200 μM did not result in any increase in the number of apoptotic cells in the brain (Fig 3D–3F and 3M). PolyR injection resulted in a similar pattern to that of the control injection with unchanged levels of TUNEL-positive cells (Fig 3G–3I and 3M). However, Trans injection led to a statistically significant and dramatic increase in apoptotic cells at the ventricle, as well as in the parenchyma (Fig 3J–3L). Upon injection, we determined a 3-fold increase in the number of TUNEL-positive cells compared to a stabbed hemisphere (Fig 3M).

**Fig 4:**
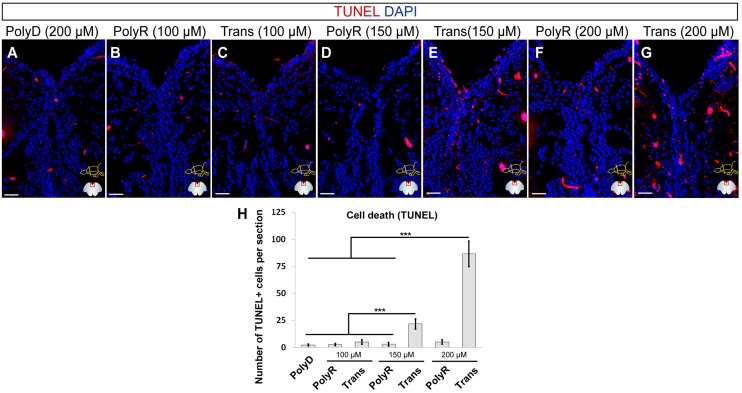
Dose-dependency of Cell Death Response. TUNEL staining on rostral telencephalon of PolyD (200 μM, A), PolyR (100 μM, B; 150 μM, D; 200 μM, F), and Trans (100 μM, C; 150 μM, E; 200 μM, G) peptide-injected brains. (H) Quantification of TUNEL-positive cells. DAPI is used for nuclear counterstaining (blue). Scale bars = 100 μm, n = 4, data are mean + s.e.m.

To investigate whether the toxicity of the peptides are dose-dependent and to find out the maximum concentration at which the peptides are not toxic, we injected the peptides at three doses of 100 μM, 150 μM and 200 μM (Fig 4A–4G). We found that compared to the PolyD control (Fig 4A and 4H), polyR did not elicit any cell death response at the doses tested (Fig 4B, 4D, 4F and 4H). Trans peptide showed increased levels of TUNEL-positive cells at 150 μM and 200 μM in a statistically significant manner (Fig 4C, 4E, 4G and 4H). These results suggest that while PolyR peptide is not toxic at such concentrations, Trans peptide can be used up to 100 μM without any cell death response.

### Trans but not PolyR peptide induces an immune reaction upon translocation at high doses

**Fig 5:**
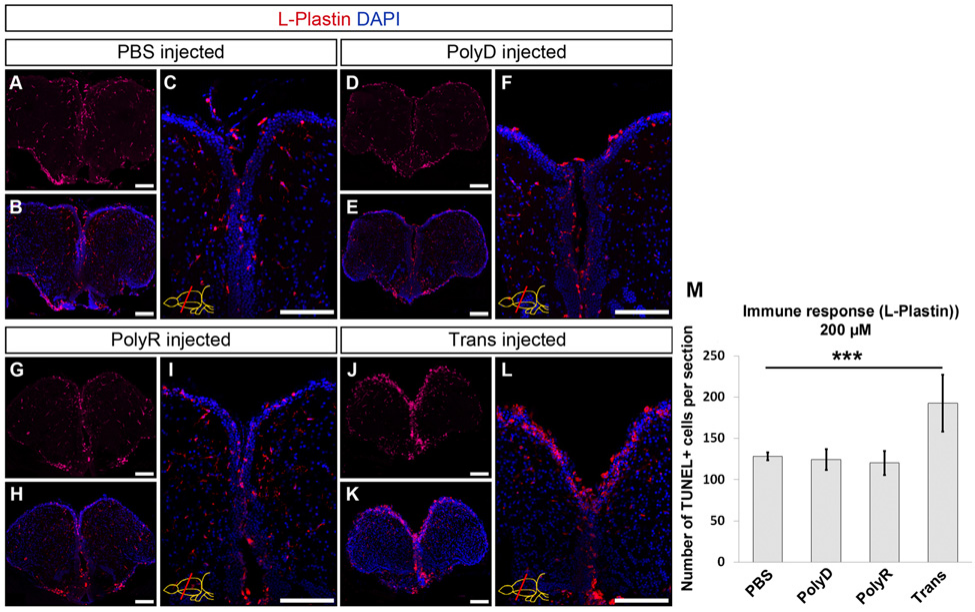
Immune Response after Peptide Injection. (A) L-Plastin immunohistochemistry (IHC) on PBS injected brain, used as control. (B) DAPI counterstaining on A. (C) Magnified medial ventricular region of B. (D) L-Plastin IHC upon PolyD injection and DAPI counterstaining (E). (F) High magnification of medial ventricular region of E. (G) L-Plastin IHC on PolyR injected brain and DAPI counterstaining (H). (J) L-Plastin IHC on Trans injected brain. (K) DAPI counterstaining on J and high magnification of medial ventricular region (L). (M) Graph depicts the quantification of L-Plastin-positive cells per telencephalic hemisphere. Scale bars: 100 μm, n = 3, data are mean + s.e.m.

The injection of the peptides might cause an immune reaction in the adult zebrafish brain, which would be an undesired situation for physiological delivery of cargo. In order to address whether peptides elicit an immune response, we determined the number of cells positive for L-Plastin, a marker for leukocytes and microglia (Fig 5). In congruence with the previously published reports [36,37], PBS-injected control brains display a consistent number of L-Plastin-positive cells at an average of 35 ±6 (Fig 5A–5C and 5M). Control peptide PolyD and PolyR injections do not alter the number of leukocytes/microglia (Fig 5D–5I and 5M), yet Trans injection causes an increase of L-Plastin staining to an average of 70 ±7 cells per hemisphere per section (Fig 5J–5M).

**Fig 6:**
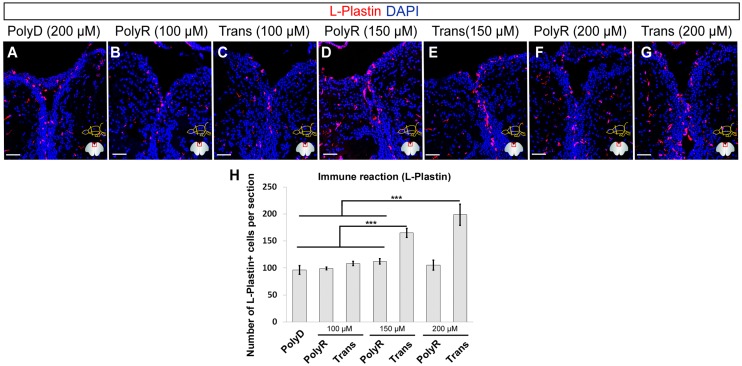
Dose-dependency of Immune Response. L-Plastin immunohistochemical staining on rostral telencephalon of PolyD (200 μM, A), PolyR (100 μM, B; 150 μM, D; 200 μM, F), and Trans (100 μM, C; 150 μM, E; 200 μM, G) peptide-injected brains. (H) Quantification of L-Plastin-positive cells. DAPI is used for nuclear counterstaining (blue). Scale bars = 100 μm, n = 4, data are mean + s.e.m.

To analyze the dose-dependency of the immune response, we injected the peptides at three doses of 100 μM, 150 μM and 200 μM (Fig 6A–6G). We showed that compared to the PolyD control (Fig 6A and 6H), polyR did not elicit any immune response at the doses tested (Fig 6B, 6D, 6F and 6H), while Trans peptide showed increased numbers of immune cells at 150 μM and 200 μM (Fig 6C, 6E, 6G and 6H). Consistent with our TUNEL results (Fig 4), these results suggest that while PolyR peptide is not causing an immune reaction at tested concentrations, Trans peptide can be used up to 100 μM without activating the immune system.

**Fig 7:**
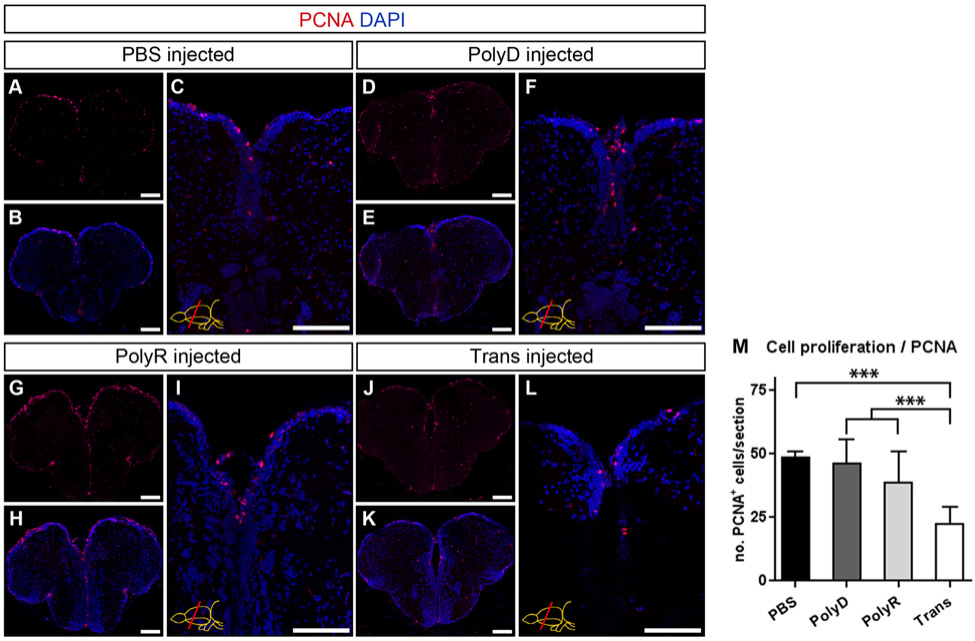
Cell Proliferation after Peptide Injection. (A) PCNA immunostaining on PBS-injected brains. (B) DAPI counterstaining on A. (C) Magnified medial ventricular region of B. (D) PCNA immunostaining on PolyD (control)-injected brains. (E) DAPI counterstaining on D. (F) Magnified medial ventricular region of E. (G) PCNA immunostaining on PolyR-injected brains. (H) DAPI counterstaining on G. (I) Magnified medial ventricular region of H. (J) PCNA immunostaining on Trans-injected brains. (K) DAPI counterstaining on J. (L) Magnified medial ventricular region of K. (M) Quantification of PCNA^+^ cells per telencephalic hemisphere section. Scale bars = 100 μm, n = 3 for every dataset, data are mean + s.e.m.

Taken together these results show that Trans peptide exerts strong cytotoxic effects at doses above 100 μM upon translocation, which correlates with a strong immune response, whereas PolyR does not display any of these unwanted side effects (Fig 4 and Fig 6). Furthermore, a quantification of ventricularly proliferating progenitor cells, as determined by PCNA immunohistochemical staining, results in an almost 50% decrease to an average 22 ± 5 proliferating cells upon Trans peptide injection at 200 μM when compared to control and PolyR injected brains, in which the homeostatic proliferation level are not altered (Fig 7). These results suggest that PolyR and Trans can be used as targeting moieties in adult zebrafish brain at an optimum concentration of 100 μM.

### PolyR can carry plasmid DNA into the brain tissue many cell diameters away from the ventricle

**Fig 8:**
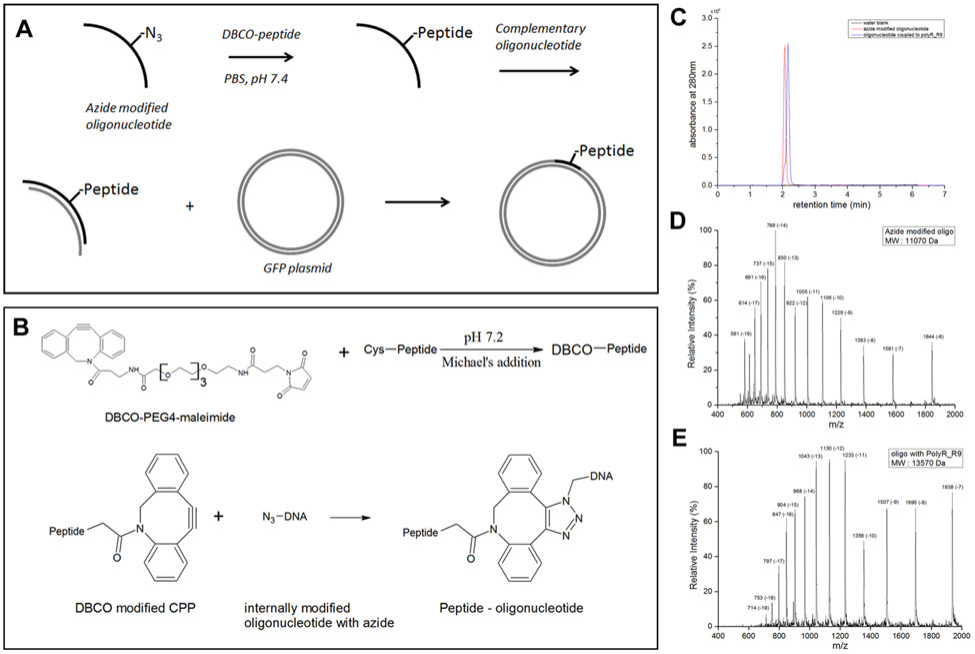
Synthesis of Peptide-plasmid Conjugate. (A) Scheme for oligonucleotide-polyR synthesis using click chemistry and incorporation into GFP reporter plasmid. (B) Reaction scheme for the coupling of Dibenzocyclooctyne (DBCO)-PEG4-maleimide to peptides using Michael-type addition, and the reaction scheme for the click reaction of DBCO-peptide to Azide-modified oligonucleotide. (C) UPLC chromatograms for the HPLC purified oligonucleotide internally modified with Azide (red) and oligonucleotide conjugated to polyR peptide (blue). (D) ESI-MS spectrum of oligonucleotide internally modified with Azide. (E) ESI-MS spectrum of oligonucleotide conjugated to polyR peptide.

To investigate if the PolyR peptide can help carry an entire plasmid into the brain tissue, we used copper-free click chemistry to prepare the construct [38,39] (Fig 8A). The PolyR peptide with a cysteine residue at the N-terminal was synthesized using solid phase peptide synthesis. The cysteine-containing peptides were conjugated to dibenzocyclooctyne-PEG4-maleimide (DBCO-PEG4-maleimide) using Michael’s addition (Fig 8A and 8B). The DBCO containing peptide was then conjugated to an oligonucleotide internally modified with an azide moiety via copper-free click chemistry. The constructs were HPLC purified and characterized using LC-MS at each stage of the chemical synthesis (Fig 8C–8E). The corresponding oligonucleotides were annealed at the same molar concentration using a programmed thermal cycler. We then integrated these DNA fragments into plasmid DNA containing a functional expression cassette with a CMV promoter and a downstream open reading frame for a GFP reporter (Fig 8A). The docking sites for the peptide containing oligonucleotides are upstream to the CMV promoter.

**Fig 9:**
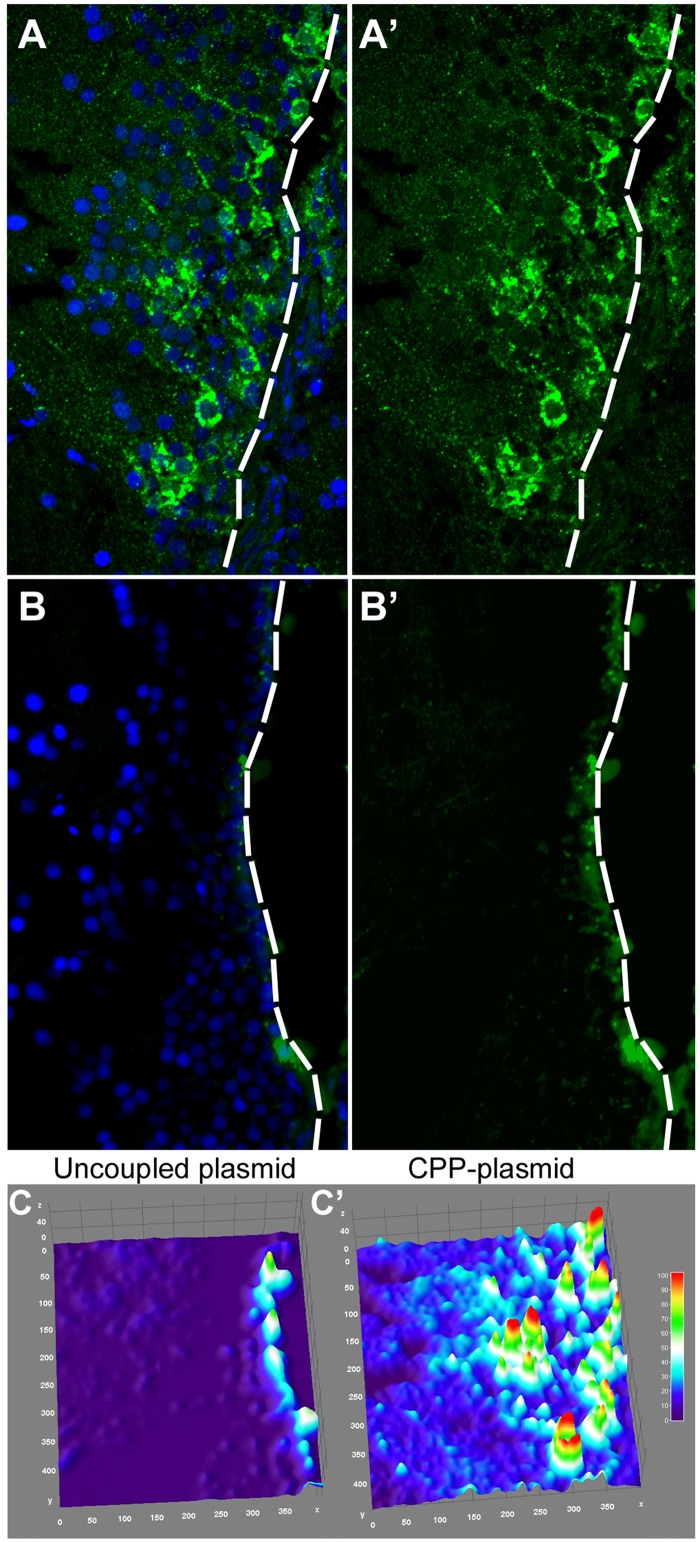
Delivery of Plasmid DNA with PolyR. (A) GFP immunohistochemistry (IHC) and DAPI staining on brains injected with PolyR-coupled GFP-expression plasmid. (A’) Green channel alone. (B) GFP immunohistochemistry (IHC) and DAPI staining on brains injected with uncoupled GFP-expression plasmid. (B’) Green channel alone. (C) Surface plot projection of the fluorescence intensity in brains injected with uncoupled plasmid. (C’) Surface plot projection of the fluorescence intensity in brains injected with PolyR-coupled plasmid.

Upon CVMI of peptide-tagged plasmid and immunohistochemical staining on the brain tissue at 1 day post injection, we observed GFP-positive cells many cell diameters away from the ventricular surface (Fig 9A and 9A’). This is not the case for injection of bare plasmid with DBCO-backbone, where GFP-positive cells are rather scarce and only present on the ventricular surface (Fig 9B and 9B’). Similarly, PolyD-control peptide coupled to the same plasmid DNA showed a GFP-expression similar to non-coupled DNA (data not shown). Surface plots indicate that significant levels of green fluorescence could be observed in deeper cells away from the ventricle (Fig 9C and 9C’). It is also worth noting that, despite significant enhancement of DNA penetration, the overall qualitative properties (size, coiled state, charge, etc.) of the peptide tag seem to influence the translocation capacity of the cell-penetrating peptides.

## Conclusions and Outlook

We demonstrate that PolyR and Trans peptides – two cell-penetrating peptides – can be used to deliver various cargos into the ventricular zone cells and deeper brain tissue in adult zebrafish brain at a concentration of 100 μM, above which the Trans peptide shows toxicity and immune response. Due to this ability, these peptides can serve as effective research tools for functional studies in adult zebrafish brain, and presumably also other zebrafish tissues which are hard to target with existing methods. Currently, the mode of internalization of PolyR and Trans peptide remains under discussion [40], but could be identified in a peptide, cargo and tissue-dependent manner. This understanding could serve as a valuable tool for diverse applications such as drug delivery, cell labeling, and misexpression of protein variants using expression constructs or gene silencing constructs. Use of these peptides in combination with existing tools, such as genetic recombineering or misexpression tools (e.g. Cre-lox, Gal4-UAS, rtTA-Tet-On/Off), or for delivering pro-drugs or other substrates, might also potentiate the applicability of these tools. Since small molecules can be delivered easily into the brain tissue, they can also be used for chemical screens. With all these properties and prospects, cell-penetrating peptides could gain wide usage for enhanced functional analyses in the adult zebrafish brain and other tissues.

## Materials and Methods

### Ethics statement

All animal experiments were carried out in accordance with the recommendations and permits of the Landesdirektion Dresden (Permit numbers: AZ 24D-9168.11-1/2008-2, 4 and 14). Surgery was performed under anaesthesia, and all efforts were made to minimize suffering. Fish were raised and kept at 28°C under a 14 hour light, 10 hours dark cycle, and fed with brine shrimp artemia daily as described in [41]. Wild-type 6–8 months old adult fish were used from the *gol-b1* line in the AB genetic background.

### Peptide synthesis, purification and analysis

For peptide synthesis all required chemicals have been purchased from IRIS Biotech GmbH (Marktredwitz, Germany) unless otherwise specified. Acetonitrile (for UPLC/LCMS), dichlormethane (DCM), diethylether, formic acid (FA), trifluoroacetic acid (TFA), triisopropylsilane(TIS) have been bought from MERCK KGaA (Darmstadt, Germany). Acetic anhydride and N-methylmorpholine (NMM) have been bought from Sigma-Aldrich Co. LLC. (St. Louis, MO, USA). Dithiotritol (DTT) from Prolab VWR International, LCC. (Radnor, PA, USA). Acetonitrile (for HPLC) was bought from TH. Geyer (Renningen, Germany). 5(6)-Carboxyfluorescein was brought from Acros Organics (Fisher Scientific Company L.L.C.). TentaGel S RAM Fmoc rink amide resin was bought from RappPolymere GmbH (Tübingen, Germany). Peptide synthesis columns and syringes with included filters were bought from Intavis AG (Cologne, Germany). Water was taken from a Milli-Q machine (Milli-Q Advantage A10, EMD Millipore Corporation, Billerica, MA, USA) with a Milli-Q filter LCPAK0001. Polytetrafluoroethylene (PTFE) filter, polyvinylidenefluoride (PVDF) syringe filter, filter holder were bought from Sartorius Stedtim (Aubagne, France).

Peptides have been prepared using standard 9-fluorenylmethoxycarbonyl (Fmoc) chemistry on a solid-phase with 2-(1H-benzotriazol-1-yl)-1,1,3,3-tetramethyluronoium hexafluorphosphate (HBTU) activation on an automated solid-phase peptide synthesizer (ResPep SL, Intavis) [25,42]. For good peptide quality, each amino acid was coupled twice, each with 5 times excess and all non-reacted amino groups were caped with acetic anhydride. Upon completion of synthesis coupling to 5(6)-Carboxyfluorescein was done manually via HBTU coupling on the resin itself. The peptide was then cleaved from the resin with TFA/TIS/water/DTT (90(v/v):5(v/v):2.5(v/v):2.5(m/v)) for 2 hours. The product was precipitated and washed with ice-cold diethyl ether.

The peptide was solved in Milli-Q water and peptide purification was performed via reverse-phase high pressure liquid chromatography (HPLC) on a preparative HPLC (ProStar, Agilent Technologies) equipped with a preparative C18 column (AXIA 100A, bead size 10μm, 250x30 mm, Phenomenex). The peptide was eluted from the column by applying a gradient from 5% to 100% solvent B over 30 min at 20 mL/min, where solvent A is 0.1% TFA in water and solvent B is 0.1% TFA and 5% of water in acetonitrile.

Purity was confirmed by analytical reverse phase Ultra-high Pressure Liquid Chromatography (UPLC Aquity with UV Detector) equipped with an analytical C18 column (ACQUITY UPLC BEH C18, bead size 1.7 μm, 50x2.1 mm) using anisocratic gradient and electrospray ionization mass spectrometry (ESI-MS) (ACQUITY TQ Detector).

The PolyR peptide with a cysteine residue at the N-terminus was synthesized using solid phase peptide synthesis. The cysteine-containing peptide was then conjugated to dibenzocyclooctyne-PEG4-maleimide (DBCO-PEG4-maleimide) using Michael’s addition (Fig 8B). The oligonucleotides with a sequence of CAT GGG GAG CGC ATG GTX GAA TGA CTC CTA CCG CG, where X is an internally azide-modified thymidine and the complementary oligonulceotide CCC TCG CGT ACC AAC TTA CTG AGG ATG were purchased from IBA Life Sciences, Göttingen, Germany. The DBCO containing peptide PolyR was covalently conjugated in an equimolar ratio, to the azide-containing oligosaccharide with copper-free click chemistry (Fig 8A). The constructs were HPLC purified and characterized using LC-MS (Fig 8C, 8D and 8E) at each stage of the chemical conjugations. The corresponding oligonucleotides were annealed at an equimolar concentration using a programmed thermal cycler. These oligonucleotides – when annealed – give two sticky overhangs compatible with KpnI and KasI restriction enzymes. The Kpn1 and KasI ligation sites were used to perform the ligation to the plasmid.

### Cerebroventricular microinjections

Cerebroventricular microinjections (CVMI) were executed as described in [7,8]. Fish were anesthetized using MESAB (0.01%) and an incision placed above the optic tectum with a fine needle (Becton Dickinson Biosciences). Through this hole a glass capillary is inserted and the liquid disperses evenly throughout the cerebroventricular fluid upon high pressure injection.

### Tissue preparation and sectioning

Fish were sacrificed 1 day post injection and heads fixed over night at 4°C in 2% PFA. Thereafter heads were transferred to 20% Sucrose-EDTA in 0.1 mM phosphate buffer, pH 7.5. For cryosectioning embedding in Sucrose(20%)-Gelatine (7.5%) on dry ice was done and heads stored at -80°C. 12 μm cryosections were cut using a cryostat microtome and stored at -20°C.

### Immunohistochemistry

Stainings were performed as previously described in [36,43]. Yet after the first washing step a 6 min citrate buffer treatment at 85°C was done. Primary antibodies were sheep anti-fluorescein AP (1:4000, Roche), mouse anti-PCNA (1:500, Dako Cyto) and rabbit anti L-Plastin (Lcp1, 1:7500, a kind gift from Michael Redd, University of Utah, Salt Lake City, UT, USA), they were diluted in PBS. They were incubated at 4°C overnight and secondary antibodies for 30 min at room temperature. Secondary antibodies were Alexa anti-rabbit and anti-mouse 488, 555 (1:500, Molecular probes). FastRed stainings were carried out using Fast Red TR/Naphtol AS-MX tablets (Sigma), according to manufacturer's instructions. Lastly sections were washed using PBSTx and mounted. All immunohistochemical stainings were done on at least five individuals.

### TUNEL assay

TUNEL stainings of sections were carried out using the ApoTag Red In Situ apoptosis Detection Kit (Chemicon International). Stainings were performed according to manufacturer’s instructions and positive and negative controls were included.

### Imaging and statistical analysis

Images were acquired using an inverted Zeiss AxioImager Z1. Only sections between the caudal end of the olfactory bulb and anterior commissure were counted. The quantification of the average translocation range was done using the AxioVision free hand surface area measurement tool. The area showing anti-fluorescein staining in the left hemisphere was measured against the total this telencephalic hemisphere, based on color thresholds against control images. The statistical evaluation was performed using GraphPad Prism (Version 6.02) for one-way ANOVA followed by a Tukey’s post-hoc test and for Student’s T-Test. Error bars shown are the s.e.m. and asterisks indicate significance according to: *: p0.05, **: p0.01, ***: p0.001. p>0.05 is considered not significant.
